# Why the “*Y*” Becomes a “Trans-*Y*”: Validation of the “Number *Y*” for Implant Height Prediction in Gender-Affirming Breast Augmentation Using Anatomical Implants

**DOI:** 10.1093/asjof/ojag049

**Published:** 2026-03-19

**Authors:** Sascha Wellenbrock, Laetitia S Chiarella, Isabel Kiehlmann, Tobias Hirsch, Charalampos Varnava, Maximilian Kueckelhaus, Marie-Luise Aitzetmüller-Klietz, Matthias Aitzetmüller-Klietz, Ulrich M Rieger

## Abstract

Breast augmentation is the most frequently performed gender-affirming surgery (GAS) for transfeminine individuals. Because of anatomical differences between transfeminine and cisgender women, implant selection requires tailored planning. The “Number *Y*” is an established method for implant height selection in cisgender patients. Since its adoption for transfeminine patients at our institution in 2016, its reliability in this population has not been validated. The aim of this study was to validate the Number *Y* in transfeminine patients, identify anatomical factors influencing implant height prediction, and propose an adjusted “Number trans-*Y*” to improve surgical planning. This retrospective study included 21 transfeminine patients undergoing breast augmentation as part of GAS. Preoperative implant height predictions using the Number *Y* were compared with actual intraoperative implant choices. Implant shapes were categorized as vertical-elliptical, circular, or horizontal-elliptical. Patient and implant characteristics were analyzed for concordance. The mean patient age was 39.62 years, BMI of 25.42, and the mean Number *Y* of 4.31, higher than reported in cisgender cohorts. Implant height prediction was accurate in 16 patients (76.19%). Discordant cases (23.81%) had significantly smaller thoracic perimeters (9 cm difference, *P* = .01) and lower Number *Y* (3.96 ± 0.11 vs 4.42 ± 0.28; *P* < .001). Most concordant cases received low-height implants, whereas discordant cases were predicted to require middle-height implants but ultimately received low-height implants. The Number *Y* has limited predictive accuracy in transfeminine patients because of distinct thoracic anatomy and higher *Y* factors. An adjusted Number trans-*Y* is proposed to optimize implant selection and surgical outcomes in this population.

**Level of Evidence: 4 (Therapeutic)**  
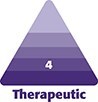

Breast augmentation is the second most performed aesthetic procedure in the world, with almost 1.89 million procedures per year.^1^ Among individuals affected by gender dysphoria re an underestimated population, affecting an estimated 25 million individuals worldwide based on census data extrapolations.^2^ Studies estimated that 20% to 40% of transgender individuals seek gender-affirming surgery (GAS); however, these estimates are based on surveys of convenience samples of transgender individuals, which limit their generalizability.^3,4^ Ha et al found that there is a 5-fold increase in GAS procedures in the last 2 decades in the United States of America.^5^ Chest feminization surgery (CFS), particularly breast augmentation with implants, is the most commonly performed GAS in transfeminine patients.^6^ Numerous studies have demonstrated the profound impact of CFS on mental health, quality of life, and social functioning.^7-10^

However, estrogen therapy alone often fails producing sufficient breast growth, with 50% to 70% of the patients left dissatisfied with their mammary profile, seeking top surgery, and requiring implant-based augmentation to attain a feminized chest.

Despite the significance of this procedure for transgender women, there are no precise statistical surveys on the number of breast augmentations performed in this specific group.^11,12^

Detecting a reliable instrument for the selection of appropriate breast implants remains one of the biggest challenges in breast augmentation even for cisgender individuals. Multiple variables must be considered to select an implant that yields a natural, proportional result.

To address this complexity, Del Yerro et al introduced the “Number *Y*,” calculated as the ratio of the thorax perimeter to the sternal notch-to-nipple distance (SNND), helping determine the optimal implant shape by considering not only the width but also the height of the implant, allowing for a more personalized and anatomically harmonious selection.^13^

In the *Y* system, the calculated Number *Y* directly guides implant height selection: lower *Y* values indicate a narrower thorax relative to breast height and therefore favor vertical-elliptic implants, whereas higher *Y* values correspond to a wider thorax and support the use of circular- or horizontal-elliptic implants. For example, a patient with a thoracic perimeter of 80 cm and an SNND of 20 cm yields a Number *Y* of 4.0, which would typically indicate selection of a middle-height (circular-based) implant within the anatomical implant range. This relationship allows the surgeon to tailor implant selection to the patient's breast dimensions and aesthetic goals.

It emphasized the significance of considering the 3 distinct somatotypes: ectomorphic (asthenic), mesomorphic, and endomorphic (pyknic), which are quantitative descriptions of the shape and morphologic features of the human body.^14,15^ These body types correspond to different breast implantation base shapes, which generally align with the contour of the torso. Del Yerro's calculation, by considering somatotypes, allows surgeons to estimate the optimal implant height, particularly useful when choosing anatomically shaped implants. By accounting for vertical proportions of the thorax, the Number *Y* provides a reproducible, objective tool for implant planning, helping to personalize and standardize decision making in aesthetic breast surgery.^13^

Nevertheless, some may question the necessity of such a tool or why it requires modification in transfeminine patients. The need for structured selection tools arises from the high variability in anatomy and the subjective nature of implant planning. In cisgender populations, the Number *Y* has shown utility in navigating this complexity.^13,16^

In transfeminine individuals the anatomical conditions differ markedly because of the effects of both testosterone-induced skeletal development and because of their exogenous estrogen intake. Histopathologically, the breast of transfeminine individuals has increased fibrous tissue with a decrease of lobular density with broader thoracic cages and denser and thicker skin.^6,17^ Furthermore, there is relatively more constricted gland tissue localized around the smaller, ovoid-shaped areola, which is more laterally positioned than in the ciswomen counterpart. Thus, surgeons often used larger implants with a predominantly horizontally elliptic-shaped orientation, mitigating the unnatural appearance of a wider cleavage, and anatomic implants are often the superior choice in transgender women because of their ability to increase lower-pole fullness in combination with a harmonic upper-pole contour mimicking natural breast shape.^18-20^

Transfeminine individuals frequently present with pseudoptosis and a somatotype that differs markedly from that of cisgender women. Whereas cisgender women predominantly exhibit a mesomorphic body type, transfeminine individuals more commonly have an endomorphic body habitus, characterized by a wider sternum and thoracic framework.

Therefore, a cis-normative application of Number *Y*, based on measurements and proportions derived from cisgender female anatomy, may not yield accurate or optimal implant selection results in transfeminine patients. Nauta et al suggested the implementation of trans-adjusted somatotype classification.^21^ Because of this fact, a more deliberate approach of surgical decision making and planning to optimize outcomes for transfeminine individuals is required.^22,23^

This study thus addresses a critical gap: does the Number *Y* system work reliably in transfeminine patients, and if not, how can it be adjusted to suit their distinct anatomical characteristics? Given the lack of published studies validating implant selection tools in the transgender population, our aim was 2-fold: first, assessing the applicability of the Number *Y* in transfeminine breast augmentation, and second, proposing an adapted version a “Number Trans-*Y*,” to enhance preoperative planning and achieve more consistent, satisfying surgical outcomes. Our rationale for using and modifying the Number *Y* is grounded in the need for reproducible, individualized planning metrics in a population where traditional reference points may not apply. This reflects our broader goal: to move beyond subjective estimations and toward standardized, evidence-based planning tools tailored to transgender patients.

## METHODS

### Breast Implant Parameters

**Figure 1.**
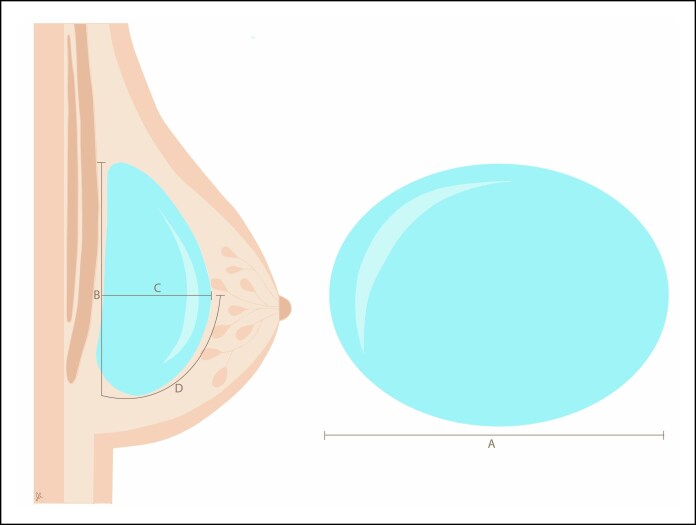
Representation of the key breast implant measurements, where A is the implant width, B is the implant height, C is the implant projection, and D is the lower ventricular curve.

Breast implant shapes are determined by several key parameters: implant base, implant projection, breast base, and implant height. The implant footprint/base, which refers to the implant's diameter and corresponds to the breast's width, can take various shapes, including vertical-elliptic, circular, or horizontal-elliptic. Implant height, on the other hand, indicates whether the implant is categorized as tall, moderate, or low. Figure 1 illustrates the key breast implant measurements and implant dimensions provided by the manufacturers: “A” represents the implant width, “B” denotes the implant height, “C” indicates the anterior implant projection, and “D” corresponds to the lower ventricular curve. As previously described, the Number *Y* provides the surgeon with control over the upper portion of the breast, reflecting how a specific implant height affects a particular breast base. Therefore, a tall implant height implies a vertical-elliptic implant shape, a middle height a circular implant shape, and a low height a horizontal-elliptic shape.

### Study Cohort

In this retrospective study, we included 25 transfeminine patients who underwent breast reconstruction as part of GAS at the Agaplesion Markus Hospital in Frankfurt, Germany, between 2016 and 2021. We analyzed preoperative implant shape predictions based on the Number *Y* method and compared them with the actual intraoperative decisions, focusing on this highly specific and relatively rare subgroup within aesthetic surgery. All transfeminine individuals asking for implant-based breast augmentation, primary or secondary, who met the guidelines of the World Professional Association for Transgender Health in its newest version and who chose anatomical implants met the inclusion criteria for the study.^24^

Patients who received a nonanatomical breast implant were excluded from the study (*n* = 4). No patients needed to be excluded because of incomplete data or inadequate follow-up. A graphical representation of inclusion and exclusion criteria is provided in Supplemental Figure 1.

### Surgical Procedure

At the hospital patients were treated aligning a standard protocol, with a confirmation of the gender dysphoria through a mental health practitioner and the initiation of hormone therapy for at least a year. Preoperatively, we examined upper thoracic anthropometrics, their soft tissue, and skin characteristics, and for each patient, the Number *Y* was calculated according to the method outlined by Del Yerro et al: Number *Y* = Thoracic perimeter/SNND.

To ensure accuracy and minimize measurement bias, all thoracic measurements were taken at the level of the inframammary fold (IMF) during the end of an unforced expiration. Subsequently, breast augmentation was performed.

All procedures in this cohort were performed by 3 consultant surgeons. Intraoperative implant size selection was therefore determined by the operating surgeon.

Implant selection was guided by a combination of preoperative anatomical measurements (including base width and soft-tissue characteristics) and patient preference. Base width and projection were determined preoperatively; however, implant height was occasionally adjusted intraoperatively to optimize soft-tissue fit and aesthetic balance. These adjustments were based on intraoperative assessment rather than predefined quantitative criteria.

In patients with tubular breast features (*n* = 3), the IMF and nipple-to-IMF distance were adjusted intraoperatively as appropriate. However, these cases were not the primary contributors to implant height discordance.

### Exposure

This study aims to evaluate the applicability of the Number *Y* in transfeminine individuals. To achieve this, the primary focus was on assessing how often the breast implant shape chosen preoperatively, based on the Number *Y*, matched the implant shape selected intraoperatively. Following Del Yerro's concept of predicting implant height, the study examined the relationship between preoperative and intraoperative implant heights and the resulting breast implant base shapes, categorized as vertical-elliptical, circular, or horizontal-elliptical. The implant shape is specifically determined by the chosen implant height, with a tall implant height corresponding to a vertical-elliptic shape, a middle implant height to a circular shape, and a low implant height to a horizontal-elliptic shape.

To further understand how differences in breast measurements between ciswomen and transfeminine individuals influence breast implant shape, patient and implant characteristics were compared. If the Number *Y*–recommended implant height did not align with the height selected intraoperatively, the match was classified as discordant; if they matched, it was classified as concordant. Subsequently, patient characteristics were collected and analyzed, with comparisons drawn between concordant and discordant implant height groups in an exploratory and descriptive manner to examine their relationship to the associated breast implant shapes.

### Statistical Analysis

Patient and breast implant characteristics were assessed for the entire cohort and compared by the exposure. Continuous variables were compared with analysis of variance if normally distributed and with the Wilcoxon rank-sum or Kruskal–Wallis test if not normally distributed. The distribution was assessed with the Kolmogorov–Smirnov test. Categorical variables were compared using Pearson's χ^2^ test.

All statistical analyses were performed using STATA16 (Version 16, STATA Corp., TX). A 2-tailed *P*-value of <.05 was considered statistically significant.

### Ethics Statement

The data presented in this study were obtained in accordance with the tenets of the latest version of the World Health Association Declaration of Helsinki. Because the data extracted from the department databases were anonymized and de-identified before release, an additional ethics statement was not required.

## RESULTS

### Overall Patient Characteristics

On average, our cohort was 39.62 years old (±15.69) with a BMI of 25.42 kg/m^2^ (±4.55 kg/m^2^). The average SNND was 21.65 cm (±1.60 cm), and the mean thoracic perimeter measured 93.17 cm (±7.51 cm). This resulted in an average Number *Y* of 4.31 (±0.32).

**Figure 2.**
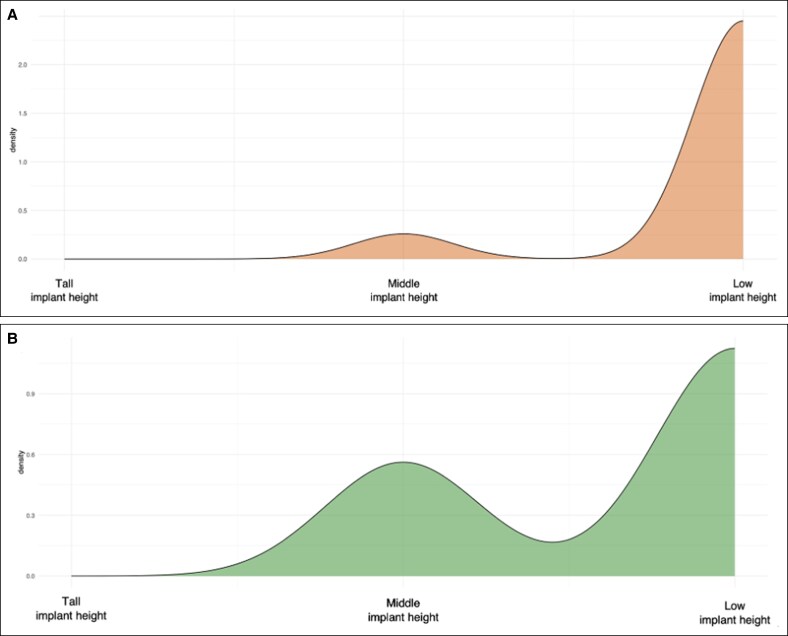
Kernel density distributions illustrating the relative frequency of implant height selection in the present study cohort. The *y*-axis represents probability density derived from kernel density estimation, reflecting the distribution of implant height categories rather than absolute patient numbers. (A) The distribution of implant heights recommended based on the Number trans-*Y* assessment. (B) The implant heights ultimately selected and implanted.

**Figure 3.**
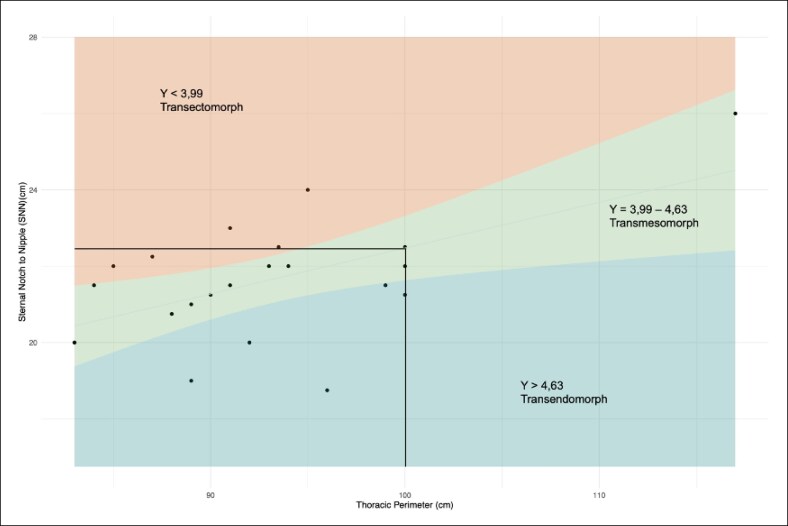
Overall regression plot illustrating the relationship between thoracic perimeter and sternal notch-to-nipple distance (SNND) used to derive the Number *Y* in the present study cohort. Individual data points represent patients included in the analysis. Shaded regions indicate the proposed Number *Y* categories (*Y* < 3.99, transectomorph; *Y* = 3.99-4.63, transmesomorph; *Y* > 4.63, transendomorph), as defined by the regression model. The vertical and horizontal reference lines illustrate the measurement thresholds applied for subgroup classification.

Most patients (62%) underwent primary breast reconstruction, with 90.48% receiving a horizontal-elliptic implant shape, followed by 2 circular-shaped (9.52%) and no vertical-elliptic-shaped implants. The overall distribution of implant shape, and their heights are represented in Figure 2, showing actual and recommended implant heights. The average Number *Y*, illustrated in Figure 3, is elevated in transgender patients undergoing gender affirmation surgery compared with cisgender women. This difference is reflected in the steeper linear coefficient with an average Number *Y* of 4.31 (± 0.32).

In further detail, the breast base averaged 134.29 mm in width (±7.79), with a mean breast height of 114.71 mm (±8.84), an anterior projection of 47.14 mm (±6.37), and a lower ventricular curve of 62.81 mm (±5.72). All implants were inserted using a dual-plane technique through a submammary incision. Patient demographics and the implant-based measurements are shown in Table 1.

**Table 1.** Patient Demographics and Implant Characteristics

	Overall cohort (*n* = 21)
Age, years	39.62 ± 15.69
SNND, cm	21.65 ± 1.60
Thoracic perimeter, cm	93.17 ± 7.51
Number *Y*	4.31 ± 0.32
Size of implant, cm^3^	349 ± 78
BMI, kg/m^2^	25.42 ± 4.55
Recommended implant height	
Tall	0 (0%)
Circular	7 (33.33%)
Low	14 (66.67%)
Actual implant height	
Tall	0 (0%)
Circular	2 (9.52%)
Low	19 (90.48%)
Implant measurements, postoperative	
Breast base, mm	134.29 ± 7.79
Breast height, mm	114.71 ± 8.84
Anterior projection, mm	47.14 ± 6.37
Lower ventricular curve	62.81 ± 5.72

BMI, body mass index; SNND, sternal notch-to-nipple distance.

### Comparison of Characteristics Between Discordant and Concordant Breast Implant Heights

Among the 21 transfeminine individuals, 16 (76.19%) received an implant height predicted by their Number *Y*, whereas 5 (23.81%) did not. Figure 2 illustrates the discrepancy between the recommended implant height and the actual implant height.

Patients with discordant implant heights were younger (31 ± 6.86 vs 42.31 ± 16.83, *P* = .16) and had a lower BMI (22.98 ± 1.72 kg/m^2^ vs 26.18 ± 4.92 kg/m^2^, *P* = .12). These differences were not statistically significant. The SNND was comparable between the 2 groups (21.75 ± 1.12 cm in the discordant group vs 21.62 ± 1.75 cm in the concordant group). However, the thoracic perimeter was significantly smaller by an average of 9 cm in the discordant cohort (86.00 ± 3.16 vs 95.41 ± 7.08, *P* = .01), resulting in a significantly smaller Number *Y* (3.96 ± 0.11 vs 4.42 ± 0.28, *P* < .001). Notably, patients with discordant implant shapes were all recommended middle implant heights with a circular implant footprint/base but ultimately received horizontal-elliptic implants. In contrast, patients with concordant implant shapes were predominantly recommended low implant heights with a horizontal-elliptic implant footprint/base (87.5%).

Anterior projection measured 44.60 ± 0.55 cm in the discordant group vs 47.94 ± 7.15 cm in the concordant group (*P* = .32). Similarly, the lower ventricular curve was 60.20 ± 1.09 in the discordant group compared with 63.62 ± 6.35 in the concordant group (*P* = .25). Further details on differences between the 2 groups are presented in Table 2.

**Table 2.** Comparison of Patient Demographics and Implant Characteristics Between Concordant and Discordant Implant Heights

	Concordant implant height (*n* = 16)	Discordant implant height (*n* = 5)	*P*-value
Age, years	42.31 ± 16.83	31.00 ± 6.86	.16
SNN distance, cm	21.62 ± 1.75	21.75 ± 1.12	.88
Thoracic perimeter, cm	95.41 ± 7.08	86.00 ± 3.16	.01*
Number *Y*	4.42 ± 0.28	3.96 ± 0.11	.002*
Size of implant, cm^3^	351 ± 90	316 ± 20	.29
BMI, kg/m^2^	26.18 ± 4.92	22.98 ± 1.72	.12
Recommended implant height			.001*
Tall	0 (0%)	0 (0%)	
Circular	2 (12.5%)	5 (100%)	
Low	14 (87.5)	0 (0%)	
Actual implant height			.43
Tall	0 (0%)	0 (0%)	
Circular	2 (12.5%)	0 (0%)	
Low	14 (87.5)	5 (5%)	
Implant measurements, postoperative			
Breast base, mm	134.69 ± 8.84	133 ± 2.74	.68
Breast height, mm	115.75 ± 9.90	111.40 ± 2.19	.35
Anterior projection, mm	47.94 ± 7.15	44.60 ± 0.55	.32
Lower ventricular curve	63.62 ± 6.35	60.20 ± 1.09	.25

BMI, body mass index; SNN, suprasternal notch-to-nipple.

^*^Significant, *P* < .05.

Although no standardized patient-reported outcome measures (PROMs) were collected, qualitative surgeon-assessed aesthetic outcomes revealed that individuals in the concordant group more frequently exhibited balanced upper-to-lower pole proportions and overall better symmetry. In contrast, patients in the discordant group occasionally showed signs of insufficient upper pole fill and flatter breast contours, which may suggest comparatively lower aesthetic satisfaction.

## DISCUSSION

Despite the abundance of literature validating breast augmentation methodologies in cisgender women, research specifically focused on transfeminine individuals remains limited. Exogenous estrogen therapy promotes breast growth initiating diverse effects on the thoracic wall and fat distribution, generally leading to noticeable changes within the first 6 months.^25^ However, the anatomic baseline of transfeminine individuals, shaped by testosterone during adolescence, differs considerably from that of cisgender women, complicating the application of cis-specific implant planning tools.

Although a minority of trans women report satisfaction with breast contour following hormone therapy alone, the majority seek surgical augmentation, thus raising the crucial question of appropriate implant selection.

Validated implant selection systems such as the Number *Y* score rely heavily on breast base width and thoracic morphometry. Yet the effects of hormonal transition blur the traditional binary classification of thoracic anatomy, challenging the direct application of cis-derived algorithms. This study is the first to assess the applicability of the Number *Y* system in a transfeminine population.

Our data demonstrated a significantly elevated average *Y* factor of 4.31, suggesting a shift toward an endomorphic somatotype, compared with the cisgender mesomorphic norm (*Y* factor ∼3.8-4.2).

Figure 3 depicts a gradual increase in the absence of tall, plenty moderate- and mostly low-height implants, different from the Gaussian distribution we expect from the cis-feminine normative population.^13^

These findings are consistent with previous studies reporting that transfeminine individuals exhibit a shorter IMF distance, a larger thoracic circumference, and a higher BMI compared with cisgender women. In our overall cohort, chest perimeter was relatively high compared with cis-female patients. However, within the discordant group, chest perimeter was lower than in the concordant group.

Despite sustained estrogen exposure, soft-tissue deficiency, especially in the upper and lower poles, remains a common challenge. These unique anatomical features necessitate individualized preoperative planning and justify the development of a trans-specific adaptation of the Number *Y* regression curve, as presented in Figure 3.^19,21^

The predominant implant shape was the horizontal elliptic with >90% of the sample underlining the anatomic consideration of a wider torso with an extended breast base and an overall bigger perimeter as seen in large comparison studies between cisgender and transgender top surgery population.^26,27^

The discrepancy in implant selection between the *Y*-concordant and *Y*-discordant subgroups highlights a limitation of the original Number *Y* system.

**Figure 4.**
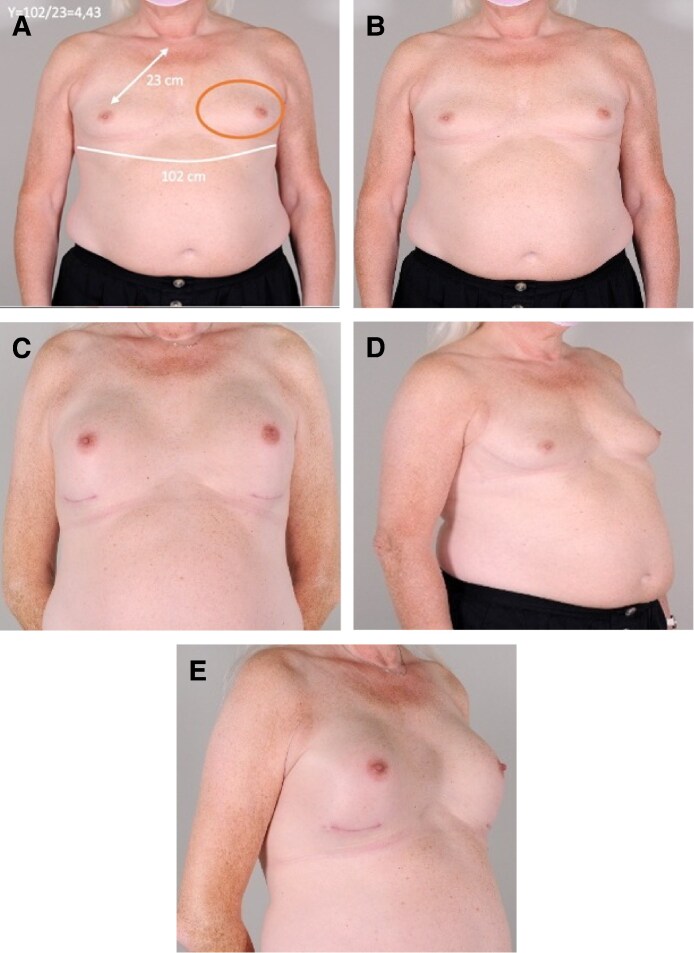
(A) Number *Y* calculation in a concordant case. Recommended and actual implant height (brown ellipse): low. (B-E) Preoperative and postoperative results of Number *Y* concordant, a 68-year-old transfeminine woman. Eight months postoperative breast augmentation with a 445 cc horizontal-elliptic base with moderate projection.

**Figure 5.**
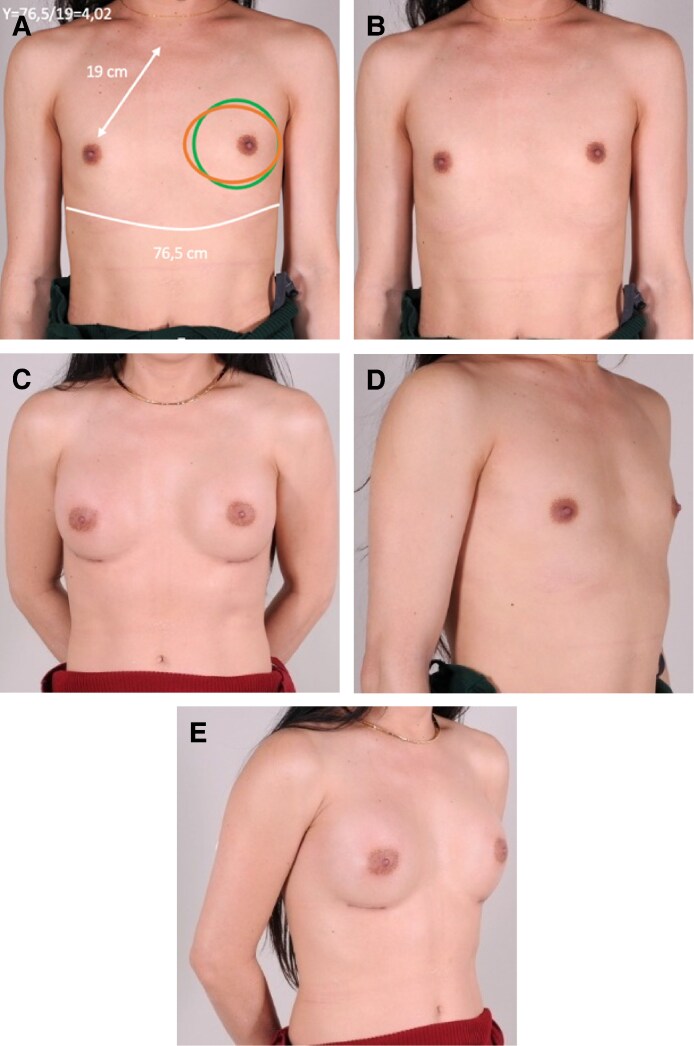
(A) Number *Y* calculation in disconcordant case, recommended implant height (green circle): moderate; actual implant height (brown ellipse): low. (B-E) Preoperative and postoperative results of Number *Y* disconcordant of a 38-year-old transfeminine woman. Twelve months postoperative breast augmentation with a 265 cc horizontal-elliptic base implant with moderate projection.

Although it reliably predicts implant characteristics for *Y* factors >4.2 (Figure 4A, B), by aligning the thoracic perimeter, SNND with the horizontal shape, respectively, it becomes less accurate for individuals with lower thoracic perimeters. In these cases, the recommended implant height failed to produce an aesthetically pleasing result, as demonstrated in Figure 5A and B, in contrast, especially in the group of the transfeminine individuals with a smaller thoracic perimeter, taking into account the anatomic circumstances in transfeminine individuals.

In the discordant group, patients had the same SNND and lower chest perimeter, yet medium-height implants were inappropriate. This reflects the limitations of these linear parameters: they fail to account for overall thoracic projection, chest wall flatness, or the degree of soft-tissue coverage. In such cases, clinical judgment favored lower-height implants to better fit the shallower chest anatomy and to avoid unnatural protrusion. As exemplified in Figure 5, a low-height implant was chosen despite the tool suggesting a medium-height option.

In the author's opinion, dealing with a smaller-sized, laterally positioned nipple–areola complex, circular-based anatomical implants were insufficient for adequately addressing the medial pole for proper cleavage. When the pocket is prepared medially, these implants fail to properly augment the lateral aspect, thereby creating inadequate lateral fullness, which is a key to improving the constricted, in many cases mildly tuberous, baseline breast anatomy.

Based on these observations, we propose a revised classification system, the Number trans-*Y*, to guide implant shape selection more appropriately in transfeminine patients. For *Y* factors ≥3.99 (reflecting trans-endomorphic and trans-mesomorphic types), we recommend horizontal-elliptic implants. For *Y* factors <3.99 (trans-ectomorphic), round or vertical-elliptic implants may offer better aesthetic outcomes.

Because anatomical implants provide a natural breast shape, their proper placement requires significant expertise and carries the additional risk of implant malrotation especially in forementioned smaller perimeters. To address this, Decuypere et al switched to using Ergonomix implants (Motiva Implants, Establishment Labs, Alajuela, Costa Rica), which are designed to adjust to the patient's position under the influence of gravity, thanks to their specialized technical features, as a potential alternative in those cases.^20^

Current literature consistently supports the notion that although transgender women tend to utilize larger implants because of anatomical factors and personal preference for feminization goals, complication rates remain comparable to those seen in cisgender women undergoing similar procedures, except for hematoma but more importantly asymmetry and mispositioning of the implant, underscoring the necessity for tailored preoperative counseling regarding implant selection and potential outcomes.^28,29^

In cases of moderate NAC asymmetry, implant selection and positioning were deliberately based on the breast with the lower NAC because this side determines the limiting factor for lower-pole expansion and implant positioning. Minor NAC asymmetries were not specifically corrected within the scope of this study. Although established surgical techniques such as differential pocket dissection or adjunctive mastopexy may be used in clinical practice when indicated, these procedures were beyond the focus of the present analysis. Consequently, procedures other than anatomical implant augmentation were examined.

This study has several limitations. First, the small cohort size (*n* = 21) and the limited geographic area reduce the generalizability of the findings to broader populations or different clinical settings. We acknowledge that this small sample size limits the statistical power of our analysis. Nevertheless, we emphasize that this represents the first formal validation of the Number *Y* system in transfeminine breast augmentation in a highly specific and underrepresented patient population and, to our knowledge, the first attempt to propose a systematic modification, the Number trans-*Y*, tailored specifically to transgender patients. Second, SNND and thoracic perimeter were obtained from different examiners (3 consultant surgeons), which may have affected the reliability of anthropometric measurements and introduced a potential degree of subjectivity in implant selection.

Third, implant height was occasionally modified intraoperatively based on the surgeon's qualitative assessment of soft-tissue accommodation and overall aesthetic harmony, rather than on predefined quantitative criteria. This inherently introduces a degree of subjectivity and potential selection bias, which should be considered when interpreting the results.

The predominance of horizontal-elliptic implants observed in this cohort may, in part, reflect surgeon-specific aesthetic preferences rather than an intrinsic limitation of the measurement system itself. Owing to the exploratory nature of the present study, the available data do not allow such potential surgeon-related bias to be definitively excluded. Further validation of the Number trans-*Y* in larger, independent cohorts, ideally incorporating blinded outcome assessment and PROMs, will be necessary to disentangle measurement performance from individual aesthetic preference and to substantiate its clinical utility.

Consequently, multicenter, prospective studies with standardized preoperative measurements and postoperative satisfaction assessments in more diverse patient populations are required to provide a more comprehensive evaluation of breast implant outcomes.

## CONCLUSIONS

In transgender patients undergoing gender affirmation surgery, our findings suggest that the original Number *Y* system, while conceptually valuable, is limited in its direct applicability because of consistently higher *Y* factor measurements compared with cisgender women. We propose a modified version of this measurement, named Number trans-*Y*, to better predict implant height in transgender patients.

## Supplementary Material

ojag049_Supplementary_Data
