# Supplementary figures and images for: Why the “Y” Becomes a “Trans-Y”: Validation of the “Number Y” for Implant Height Prediction in Gender-Affirming Breast Augmentation Using Anatomical Implants
Source: Aesthet Surg J Open Forum. 2026 Mar 19;8:ojag049. doi: 10.1093/asjof/ojag049 (PMC13082374; doi:10.1093/asjof/ojag049)

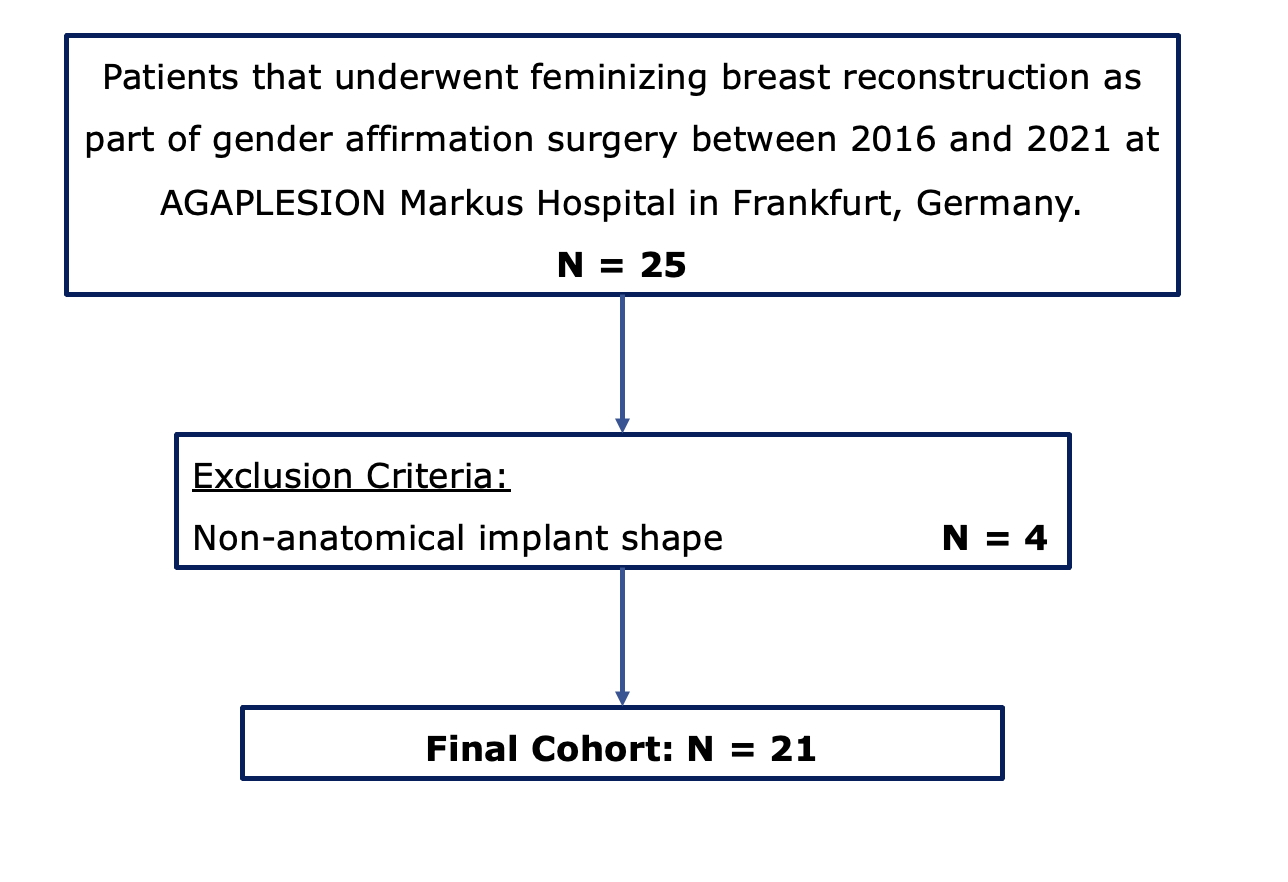

Supplement: ojag049_Supplementary_Data [file ojag049_supplementary_data.zip › Supplement Figure 1.jpg]
